# Nanomaterial Complexes Enriched With Natural Compounds Used in Cancer Therapies: A Perspective for Clinical Application

**DOI:** 10.3389/fonc.2021.664380

**Published:** 2021-04-01

**Authors:** María Zenaida Saavedra-Leos, Euclides Jordan-Alejandre, César López-Camarillo, Amaury Pozos-Guillen, César Leyva-Porras, Macrina Beatriz Silva-Cázares

**Affiliations:** ^1^ Coordinación Académica Región Altiplano, Universidad Autónoma de San Luis Potosí, Matehuala, Mexico; ^2^ Posgrado en Ciencias Genómicas, Universidad Autónoma de la Ciudad de México, Mexico City, Mexico; ^3^ Laboratorio de Ciencias Básicas, Facultad de Estomatología, Universidad Autónoma de San Luis Potosí, San Luis Potosi, Mexico; ^4^ Laboratorio Nacional de Nanotecnología, Centro de Investigación en Materiales Avanzados S.C. (CIMAV), Chihuahua, Mexico

**Keywords:** nanomaterials, cancer, quercetin, resveratrol, pharmacological repurposing

## Abstract

Resveratrol and quercetin are natural compounds contained in many foods and beverages. Reports indicate implications for the health of the general population; on the other hand the use of both compounds has interesting results for the treatment of many diseases as cardiovascular affections, diabetes, Alzheimer’s disease, viral and bacterial infections among others. Based on their capacities described as anti-inflammatory, antioxidant, and anti-aging, resveratrol and quercetin showed antiproliferative and anticancer activity specifically in maligned cells. These molecular characteristics trigger the pharmacological repurposing of both compounds and improved its research for treating different cancer types with interesting results at *in vitro, in vivo*, and clinical trial studies. Meanwhile, the development of different systems of drug release in specific sites as nanomaterials and specifically the nanoparticles, potentiates the personal treatment perspective in conjunct with the actual cancer therapies; regularly invasive and aggressive, the perspective of nanomedicine as higher effective and lower invasive has gained popularity. Knowledge of molecular interactions of resveratrol and quercetin in diseases confirms the evidence of multiple benefits, while the multiple analyses suggested a positive response for the treatment and diagnostics of cancer in different stages, including at metastatic stage. The present work reviews the reports related to the impact of resveratrol and quercetin in cancer treatment and its effects when the antioxidants are encapsulated in different nanoparticle systems, which improve the prospects of cancer treatment.

## Introduction

**Figure 1:**
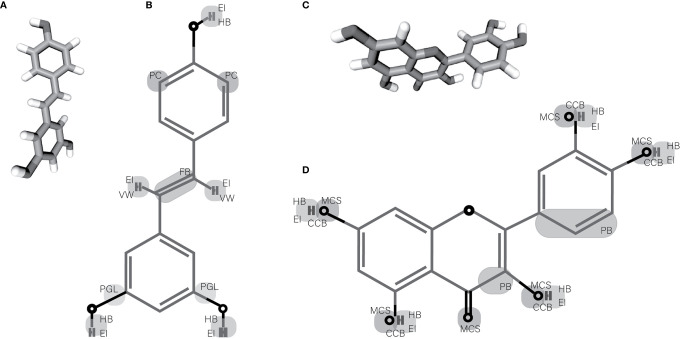
Chemical structures of resveratrol and quercetin. **(A, C)** 3D structure of resveratrol and quercetin respectively. **(B, D)** Showing the probable chemical groups in resveratrol and quercetin respectively that are susceptible to changes and that allow its complexing with nanomaterials. EI, Electrostatic Interaction; HB, Hydrogen Bound; PC, Possible Changes; VW, Van der-Waals Force; FB, Flexible Bound (Stoichiometric Changes); PGL, Possible Group Lost; MCS, Metal-Chelating Sites; CCB, Coordinate Covalent Bound; PB, Possible Break.

Cancer is a compound collective of multi-factorial diseases brought about by intracellular environmental both for aspects such genetic, epigenetic, metabolic deregulations, and other risk-factors (1, 2). This complex of diseases arises from many variations at the molecular level that produce deregulations in molecular pathways, intermediary molecules, and losing control in distinct pathways, stimulating tumorigenesis, associating it with lifestyle, longevity, and other risk factors related to contemporary life. However, palaeontological evidence revealed cancer in fossil remains up to 1.98 million years (3, 4). Nowadays, cancer has grown into the public health problem of universal attention because the treatments’ high cost and its invasiveness have generated several types of research on different drugs and modern treatments, fewer invasive and more efficient (5, 6). The progress of technologies such as nanomedicine (Nm) has opened new scopes for the treatment of many diseases (7). By definition, Nm serves to improve the bio-distribution and *in situ* drug release to deal with a specific disease, specifically in pathologies whose strategies are invasive and non-specific. For example, it could improve the release of chemotherapeutic drugs in the therapy of cancer and could increase the balance between the effectiveness and toxicity of the drugs (8). In this context, the application of different nanomaterials in the form of nanoparticles (Nps) has attracted attention because of the multiple advantages that are presented, such as low manufacturing cost, the delivery of drugs to specific sites, less invasive therapies, and greater efficiency in the treatment and recovery (9). Nowadays, cancer treatment comprises the application of drugs and chemotherapy, along with surgical intervention (10). For cancer therapy, alternative drugs as resveratrol (Rv) and quercetin (Qr) have shown potential results in their treatment, since their chemical characteristics allow them complexing with different Nps increasing their release (11, 12) (**Figure 1** and **Table 1**).

**Table 1:** Examples of chemical interaction between Rv and Qr with different nanoparticles.

Nanomaterial example	Chemical interaction	ref
PEG	Rv-OH—OH-PEG	(13)
Chitosan nanoparticle	Rv-O—H-NH_2_-chitosan	(14)
Graphene Oxide (GO)	GO=O—HO-Rv	(15)
Silver nanoparticle	Qr=O—Ag—O=Qr	(16)
Iron oxide nanoparticles	Qr-HO—H-Gly	(17)
Graphitic carbon nitride nanoparticles	Qr-HO—H-NH-	(18)

The present work reviews the perspectives and advances in implementing these drugs and its complexing with nanomaterials for their use in cancer treatment.

## Current Drugs Implicated in Cancer Treatment

Nowadays, the application of chemotherapeutic drugs is a conventional treatment perspective, where each treatment must be specific for the type of cancer (19, 20). Constant research on new drugs and new prospects has brought about novel points of view with respect to the current cancer therapy (21). For example, Zhou et al. conducted a clinical phase 3 trial in which they tested the response of lung cancer (LC) patients to Atezolizumab (Az) plus chemotherapy, showing that this combination led to patients having more prolonged survival and shorter survival likelihood of relapse (22). In another study, the effect of Lutetium-177-Dotatate, an experimental drug in phase 3 for the therapy of neuroendocrine tumors, showed that the participants treated with Lutetium-177-Dotatate showed further survival rate; besides, the participants showed a higher progression-free survival (PFS) in comparison with the control group treated with high doses of Octreotide LAR (20 to 30 mg) (23). The Olaparib impact in subjects with human epidermal growth factor receptor type 2 (HER2) negative breast cancer (BC) diagnosis showed important benefits, and the data suggested that the application of Olaparib leads to PFS for a more extended period of 2.8 months, while the risk of disease progression was 42% lower compared to monitored control groups (24). The same research group showed the PFS in BC patients treated with Olaparib compared to chemotherapy treatment, showing well toleration at the secondary effects at the studied population. Besides, they proved that Olaparib treatment didn’t show cumulative toxicity on prolonged treatment (25). Recently in 2020, Sternberg et al. demonstrated that Enzualtamide in combination with androgen deprivation for therapy of castration-resistant prostate cancer (PC) improved the mean survival and showed that the probability of death in the experimental group was 27% lower compared to the control group (26). In a prospective study conducted in 679 patients, Cremonili et al. determined that the effectiveness of FOLFOXIRI–Bevacizumab in the therapy of colorectal cancer (CRC) turns out to be better efficient in the treatment of cancer compared to patients treated with chemotherapy (27). Li et al. used *Helicobacter pylori* plus vitamin and garlic supplements to treat gastric cancer (GC); statistical data showed the reduction of the risk of death from GC and a lower incidence of GC (28). Other approaches have focused on the treatment with naturally occurring compounds; for example Stancioiu et al. showed the efficiency of spirulina, curcumin (Cm), and *Boswellia* in the treatment of thyroid nodules; the placebo-controlled study counted 34 patients treated during three months showing that the use of natural compounds reduced the nodule size (29). Many researchers demonstrated the efficiency of Rv and Qr use for multiple diseases’ treatment. Gal et al. showed that improving red blood cell aggregation using Rv in conjunct with exercise improved the hemorheological properties (30). In Alzheimer’s disease with Rv modulation of neuro-inflammation, they showed the regulation of genes related to neurodegenerative disorders as sirtuin 1 (31). Glycemic and HDL-cholesterol levels could be controlled by Rv regulating genes as PPAR-*γ* and sirtuin 1 in patients with type 2 diabetes mellitus and coronary heart disease improving the metabolic status of patients after 4-weeks of treatment, notwithstanding the study has some limitations but suggested a relevant effect of Rv in type 2 diabetes mellitus patients (32). Most et al. demonstrated the Rv impact in fat oxidation mediated by the 12-week treatment with Rv; the results suggested the modification in the microbiota of patients, specifically in men; this data suggested a positive effect in fat oxidation and mitochondrial oxidative capacity. Nevertheless more studies are necessary for understanding the correlation between gender and therapy effect (33). Another study analyzed 85 patients with stable coronary heart disease. The study reported improvement of the treatment with *ß*-blockers, statins, aspirin administrated with Qr as adjuvant, proving the cardioprotective properties of Qr at different levels (34). On the other hand, the use of Qr for treating different pathogens was analyzed in the recurrence of HPV. 59 patients were treated for 551 days, proving that the treatment showed 100% patients were HPV free. The analysis of results did not show adverse experiences in symptomatic patients; in contrast, the results weren’t replicable in asthma patients; for these reasons more studies are necessary (35). Another Qr application is the control of metabolic diseases. The Qr supplementation limited *Citrobacter rodentium* colitis effects, consistent with other studies that have shown an impact in the modification of microbiota and in the inflammatory process. Nevertheless, *Citrobacter rodentium* is a non-human pathogen, but the data suggested the possible Qr use for glut pathogen treatment (36). Likewise, Qr treatment improved the morphology and histopathology in testis. The treatment increased plasma and testicular testosterone concentrations, suggesting that Qr could prevent the toxic effects induced by sodium arsenite (37). Notwithstanding, pharmacological reuse of Rv and Qr for cancer treatment has increased since researchers have shown, among other effects, their anti-inflammatory, antioxidant, and anticancer effects.

### Perspectives of Resveratrol in Cancer Treatment

Using Rv as drug in cancer treatment has been studied. Rv is a natural polyphenolic stilbene produced by certain plants with antioxidant, anticancer, anti-inflammatory, and anti-aging properties (38–41). The Rv investigation for cancer treatment confirmed these properties in different pathologies like rheumatoid arthritis, Alzheimer’s disease, type 2 diabetes, allergic rhinitis, and cancer (31, 42–44). Researchers have studied the uses of Rv for cancer therapy. Banaszewska et al. reported Rv’s impact on the reduction of the ovarian and adrenal androgens in the treatment of polycystic ovary syndrome (POS) (45). Another clinical trial published showed Rv’s effect on reducing Vascular Endothelial Growth Factor (VEGF) and Hypoxia-Inducible Factor 1-alpha (HIF1) expression in granulose cells in women with POS. The study showed a relation between the protective effect of Rv in the patients and changes in sexual hormone levels probably by the angiogenesis pathway modification related to the VEGF and HIF1 expression levels; besides, the study suggested that the Rv consumption leads to a high-quality of the oocyte and embryo rate  (46). The analysis of phytotherapeutic approach in preventing PC relapse by applying turmeric, Rv, green tea, and broccoli sprout reported in a pilot study, revealed the use of phytotherapeutics as a workable approach to prevent a relapse of PC and as a potential treatment in men with biochemically recurrent PC, and a moderate increase rate in serum prostate-specific antigen (47). Rv has been examined in BC treatment in a group of female mice (C57BL/6) that received a special diet plus Rv; the treated group developed tumors with a smaller size in contrast with the control group; likewise the Rv presence *in situ* indicated a protective bringing about in the BC development (48). Conversely, other reports indicated that Rv can induce BC apoptosis *via* p53 by the phosphorylation of Ser-15 and that effect can masked by the dihydrotestosterone in these cells by the receptor competition; however, the result also suggested a protective role induced by the induction of apoptosis mediated by Rv (49). Other investigations treated CRC cells with Rv in conjunct with pharmacological inhibitors. The data exposed that Rv affects cell phenotype, suppressing invasion, while the viability reduction of cells contrasted to pharmacological inhibitors. Associating the effect with the Sirt1 up-regulation, FAK down-regulation, and with the inhibition of focal adhesion, Rv use showed the NF-*κ*β inhibition pathway by suppression of molecule intermediates involved in invasion, metastasis, and apoptosis (50). Another investigation exhibited the anti-tumor effects in the CRC cells of Rv. The authors showed that Rv application in conjunct with MALAT1 lentivirus short hairpin RNA, inhibited the Wnt/*β* catenin signal pathway by the interference in the gene expression of targets such as c-Myc, MMP-7, and MALAT1 (51).

### Perspectives of Quercetin in Cancer Treatment

Qr is a naturally occurring flavonoid found in a wide variety of fruits as watermelon, cantaloupe, avocado (contains one Qr unit/piece), blueberries (96 units), and Apples (24–58 units), vegetables as tofu, squash, and corn (one unit), yellow onions (326 units), and green beans with (25 units), beverages as tea, Lipton (26 units), and wine containing (8.4 units) (52). Qr is investigated for its biological activity as antioxidant, anti-inflammatory, and anticancer molecule. Besides, the antiproliferative and pro-apoptotic Qr activity is unique in cancer cells (53–55). *In vitro* research reported the improvement of apoptosis induction in human melanoma cells treated with Qr in comparison with tamoxifen (Tx). The use of 3 µM of each component triggers an important change in the clonogenic capacity of M10, M14, and MNT1 cell lines, likewise the ability of Qr to promote cell apoptosis by the heat shock protein-70 (Hsp70) down-regulation. The data suggested Qr and Tx protective role in melanoma, indicating the potential effect of both drugs in cancer treatment (56). Nowadays, many strategies result in dose-dependent toxicity as doxorubicin (Dx), being a common hazard to continuing therapy, specifically in drug-resistant cancers. In liver cancer (LiC), the protective role of Qr in treating toxicity in mice models revealed that Qr potentiated the anti-tumor effect of Dx dose-dependent toxicity and protected normal cells from the toxicity generated by toxic therapy. The accumulation of p53 mediated the Qr anti-tumor effect in LC cells, likewise the subsequent activation of mitochondrial apoptosis by the cleavage pro-caspases. The Qr use for toxicity treatments could protect the normal cells of the patient and could improve therapy resistance (57). Recently the Qr effectiveness as an adjuvant in the therapy of advanced pancreatic cancer (PcC) and other cancers types was demonstrated. Gemcitabine (Gc) use in PcC is frequent, but normally drug resistance development is the source of chemotherapy failure. The therapy, enriched with Qr induced apoptosis, causes the cell arrest in the S phase and increases the p53 expression; besides, the boosted Gc effect by Qr, especially in cancer cells resistant to Gc, was observed (58). Furthermore, the evidence indicated Qr’s anticancer role in PcC resistance to Gc treatment, presenting a relation between the high mortality rate and the receptor for advanced glycation end products and its role in different signaling cascades. They tested autophagy stimulation in PcC cell lines resistant to Gc treated with Qr as an adjuvant. The results implied the mediation of the autophagy effect by the deletion of advanced glycation end products which conduced to the increase in the ratio of Bax/Bcl-2 and the down-regulation in NF-*κβ* p65 expression, unleashing the CASP3 dependent apoptosis in the cell lines studied (59). Other reports indicated the absence or low Qr toxicity in treated rats with PC. The data showed that a dose of 30–3,000 mg of Qr/kg during 28 days did not show secondary effects at the experimental groups, demonstrating that Qr can generate chemo-protection *in vivo* models by down-regulation in oncogenes related to cell survival and a regulation in proteins related with apoptosis signaling (60). Furthermore, Qr could be employed as a preventive therapy in BC in female ACI rats by providing an enriched food with a dose of 2.5 g/kg of Qr for eight months. The rats fed with Qr and estrogen 17*β*-estradiol showed a higher PFS rate in comparison with the rats powered only with estrogen 17*β*-estradiol. The survival rate in the fed group of Qr plus estrogen 17*β*-estradiol was lower compared with the group powered only with Qr (61). A clinical trial, focusing on the gene expression of resistin in women with weight problems and POS, tested the Qr impact at metabolic and hormonal levels. A daily consumption of 1,000 mg of Qr during 12-weeks can down-regulate the resistin gene, decreasing plasma levels of POS. Qr could modulate the expression of 3*β*-hydroxysteroid dehydrogenase, Cytochrome P450 11A1, Cytochrome P450 17A1, 17*β*-hydroxysteroid dehydrogenases, androgen production, among others (62). A phase I and II study showed the safety, tolerability, and dosage determination of muscadine grape processed (known as MPX, comprising 1.2 mg of ellagic acid, 9.2 μg of Qr, and 4.4 μg of trans-Rv in 500 mg tablet) in recurrent PC cases. 4,000 mg per day was the highest safety dose and the plasma levels were undetectable. The principal secondary effects reported were flatulence, soft stools, abdominal distension and eructation. However, the treatment was tolerated during 19.8 months, while most of the patients did not show relapsed during the treatment (63). Wilms et al. reported genetic polymorphism that affected the ingestion of Qr. During the study, the volunteers ingested 1 L of blueberry and apple juice per day with a content of 97 mg of Qr per liter for four weeks. Only one of 34 polymorphisms analyzed appeared influenced in Qr metabolism mediated by the NQO1 gene expression, resulting in better metabolism of Qr. Furthermore, they viewed the Qr antioxidant role on *ex vivo* models is highly beneficial in the stressed subjects (64). Pharmacokinetics of trans-Rv and Qr were reported in a combinatory dietary. Researches dosed 2,000 mg of trans-Rv and 500 mg of Qr plus a small amount of alcohol (5% in 100 ml) during breakfast; this concentration ratio didn’t affect the pharmacokinetics of drugs. They showed the toleration of 2,000 mg of dosage by healthy subjects presented diarrhea as principal secondary effect (65).

## Nanoparticles and its Use in Cancer Treatment

In past years the Nm experimented an exponential growth, generating new approaches in the treatment of several diseases including different types of cancer (66). The progress generated by the research has generated remarkable progress in synthesis, fabrication, and characterisation of Nps specifically as liposomes for intravenous administration (67, 68). Nps can form complex with polymers, metals, and inorganic materials for controlling the delivery efficiency and effective properties. NP synthesis with indocyanine green core, coated with poly (lactic-co-glycolic acid) and cancer cell membrane has impacted the *in vitro* and *in vivo* models; the treated groups with the system plus laser triggered cell lysis. The therapy inhibited tumor growth 6 days after starting the treatment. and the survival rate was of 40%; likewise it showed a higher biomimetic rate and the ability of the system in theranostic (69). Perspectives in the BC treatment analyzed the activity of nano carried lipids with supphoraphane and Tx, considered as the “gold line”. In some BC treatment molecular subtypes showed that the system potentiated the Tx effect and decreased drug toxicity. This is a regular trouble for quality patients’ life and improves the binding affinity of Tx to estrogen receptors (70). A phase 2 clinical trial analyzed the use of nanoparticle albumin (Am)-bound-paclitaxel (Px) in complex with Gemcitabine–Cisplatine (GcCs) in advanced biliary tract cancer therapy. The typical therapy resulted in an average PFS rate of 8 months and overall survival of 11.7 months. Notwithstanding, this approach prolonged the average PFS rate during 14.9 months, and 58% of participants reported adverse events (71). In another study, Nps of Cm with serratiopeptidase showed that 95% of the complex could release the drug after 24-h at physiological conditions. Different cytotoxicity rates upper in HeLa cells (IC50 31.25 mg/ml) than MCF7 cells (IC50 0.7 mg/ml) were observed; besides this complex can potentiate the anti-tumor activity of drugs and induce the apoptosis in different cell lines (72). Different cell lines (LNCaP-AI, PC, MCF-7, BC, and QSG-7701 normal-like) showed a greater response to Dx into Np based on silica enriched with CaCO_3_. The results showed an accumulative release of Dx into pH 7.4 with 4 μg/ml, sufficiently to induced apoptosis in 31.52% of cells. The effect of the system at *in vivo* models had tumor growth and progression rate lower. The tumor weight reduction was 71% in comparison with control groups (73). Photo-thermal therapy was based in a system of polypyrrole with camptothecin-conjugated hyaluronic acid (HA) shell. The Np therapy plus chemotherapy and immunotherapy induced cell death in 4T1 cells, while the therapy in female Balb/c mice models showed the detection *in situ* 4-h post-treatment; besides, increased of CD8+ T cells, CD3+ T cells, the tumor necrosis factor α, interferon-*γ*, interleukin-1β, -2, -4, -6, and -10 (74). A transformable size/charge Np type-Trojan-Horse, integrating Dx and pheophorbide showed that increasing the temperature after laser irradiation in oral squamous cell carcinoma 3 was sufficient to induce DNA damage by the Reactive Oxygen Species (ROS) generation, and the acid pH improved drug release. The *in vivo* models showed nanoparticle accumulation *in situ* 24-h post-injection (75). A phase I clinical trial with NPs tested in patients with advanced cancer non-responsive to standard-of-care treatments demonstrated the efficiency of microRNA-34a in complex with liposomal Nps by inducing cell damage after 93 mg/m^2^ treatment. The half-life of the system was about 35 h with a tumor reassessment in 75% of the participants, while 68% showed a better expression of genes related with the tumor immune response. Notwithstanding, more of 50% participants experimented different adverse events (76). A prospective phase I/II study tested the impact of four different treatment profiles. Profile A: 150 mg/m^2^ of Am Np bound Px and 1,000 mg/m^2^ of Gc. Profile C: 125 mg/m^2^ of Am Np bound Px and 1,000 mg/m^2^ of Gc. Profiles B and D were carried out varying the drugs’ concentration for three weeks, plus a week off treatment. The results demonstrated a better cycle for each treatment profile. For profile A, the better cycle was 1.5, for profile C was 2.5, while for profiles B and D were 1.5 and 3.5, respectively. The less toxic treatments and better-tolerated profiles were B and D, which in consequence were used in the phase II study. The patients treated with these profiles presented a partial response. 65% of the patients treated with profile B showed a better disease control rate, while those treated with profile D showed a disease control of 72%. The PFS rate for each profile was 5.4 and 6.6 months, respectively (77). Another phase II clinical trial performed in metastatic castration-resistant PC patients, consisted in docetaxel (Dc) into Np with functionalized surface for targeting prostate-specific membrane antigen. Results showed a decrease of 50% in prostate specific antigen rate compared with the baseline. On the other hand, only 14.2% of patients responded to treatment, 11.9% patients had a partial response, and 21.4% patients had stable disease. The PFS rate was 9.9 months (78). On the other hand, Am-bound-Px Np plus Az in the treatment of BC patients without prior treatment with Az during 12.9 months triggered the improvement of PFS during 7.2 months in the treated group. Furthermore 7.1% of patients presented a complete response to treatment, and 5% of participants presented adverse effects, showing a high Np safety rate (79). Another report studied the impact of epirubicin (90 mg/m^2^), cyclophosphamide (600 mg/m^2^), and nanoparticle Am-bound-Px (125 mg/m^2^) in BC patients, with HER2 positive, hormone receptor positives (HR+) or TN diagnostic; 47.5% of patients treated conserved the breast; besides, 72.5% of the participants concluded the therapy with common adverse event in 55% patients, showing a higher response and better prognosis; likewise the genomic analyses showed a relation between genetic signatures and the therapy response (80). In a contradictory way, the survival rate in patients with hepatocellular carcinoma (HCC) in a phase 3 study randomized in three different groups: 30 mg/m² group (treated with 30 mg/m² Dx-loaded Nps), 20 mg/m² group (treated with 20 mg/m² Dx-loaded Nps), and control group (standard care), showed that the median survival have no statistical differences. suggesting that the therapy with Dx-loaded Nps was not effective in the control of HCC (81).

### Resveratrol Nanoparticles in Cancer Treatment

Using Nps is useful in biological applications, especially in the implementation of certain natural compounds such as Rv, **Table 2**.

**Table 2:** Examples of different nanoparticles loaded with Resveratrol and their impact in the control of cancer.

Model	System	System size	Apoptosis induction by	C.P	T.G.	Ref.
Human colorectal cancer	Lipid matrix	1000 ± 1.8 nm	Casp3	79.6%	31.4%	(82)
Human neuroglioma	Poly(d,l-lactide-co-glycolide) lipid covalently conjugated with folic acid and indocyanine green	104.5–121.1 nm	N.R.	18.6%	7.1%	(83)
Pancreatic cancer	Albumin plus human serum albumin	120 ± 2.6 nm.	Pyknotic nuclei formation.	15%	N.V.T.	(84)
Human Ovary cancer	Gemcitabine-silver	20 nm	Free radical generation, DNA damage and CASP3 and CASP9.	40%.	20%.	(85)
Breast cancer (Triple-negative)	Oxidized mesoporous carbon	240.1 nm	PAPR cleavage and activation of CASP3.	50%	N.R	(86)

C.P., Cell Proliferation; T.G., Tumor Growth; N.R., Non-Reported; N.V.T, Non-Visible Tumor.

**Table 2** summarizes some examples reported about the using of Rv Nps in the treatment of different cancer types. In 2018, Peñalva et al. determined the increment of bioavailability of Rv complex with casein Nps. The report described the release of Rv into physiologic pH and showed that the 100% of release efficiency happened at gastric fluid pH after 9-h; besides, the data were similar in Wistar rat models; pharmacokinetics data reported the half-life of about to 2.7-h, *i.e.*, ten times higher accumulation contrasted to another distribution procedure; also, they demonstrated that the excretion of the system was 48-h post-oral administration (87). A study showed that the distribution of Rv into mesoporous silica Nps in PC has 100% of distribution into pH 7.4, 8-h post-administration; the therapy diminished PC3 cells that proliferate with 20 µM, notwithstanding the IC50 was 14.86 µM; besides, the use of this system plus Dc showed an increase of 50% in the cytotoxicity in immune cells to Dc (88). In another 2018 study, the Rv used as adjuvant to omega-3 polyunsaturated fatty acids encapsulated in a lipid matrix showed 25% minor oxidation rate in rat models; the complex integration in the HT-29 CRC cells was 277% greater, and the cell growth inhibition-rate improved at 72-h post-treatment in different adenocarcinoma cells lines; besides, the CASP3 activation in cells was 150% higher compared with the control groups. The cell proliferation in the treated group was 20.4% lower in contrast with the controls (82). The brunt of Rv-ferulic acid was carried on chitosan coated folic acid into solid lipidic-Nps into apoptosis induction; they disclosed the drug-release was 42.87%; meantime the IC50 was detected around 10 µg/ml; besides the induction of apoptosis was sharper in HT-29 cells and NIH 3T3 cells evaluated with the compound in relation with non-treated groups (89). An *in vivo* research analyzed the impact of Rv and Dc encapsulated in lipid-polymer hybrid Nps conjugated with epidermal growth factor (EGF); the pharmaceutical co-delivery *in vitro* was 90% in HCC827 cells. Contrarily, the results of co-release in HUVEC cells did not show a difference between the treated group and the control group, Furthermore, *in vivo* models showed the localization *in situ* 48-h post-application in tumor tissue, leading to smaller tumor and a tumor growth rate of 79% lower in comparison with the control group (90). In 2016, a study analyzed the impingement of Rv-gold Nps complex in MCF-7 (BC cells) in invasive process prompted by 12-O-tetradecanoylphorbol-13-acetate. The therapy with 10 µM of the complex inhibited the cellular migration and invasion, seemingly by the block of NF-kB phosphorylation and the successive activation of MMP-9 and COX-2, the molecules required in the metastatic cancer process (91). In Nps of Rv loaded in nano-capsules in melanoma mice model, *in vitro* results showed that 100 µM and 300 µM decreased the cell viability of B16F10 cells between 24 and 72-h post-treatment; besides, the studied mice produced tumors smaller, 10 days post-therapy in contrast with the control group (92).The application of Nps into the theranostic procedure has effective impact in the employment of Human neuroglioma with down toxicity (<10%); the system supplemented with Rv, showed the induction of apoptosis in 81.4% of the treated cells; besides, their results showed greater targeting in tumor cells 5-min post-treatment, and it improved the localization *in situ* after 6-h post-treatment (83). On the other hand, Lv et al. reported the manufacture of a micro-bubble structure capable of discharging Rv in an exact pH; it was explained that the capability of this system to deliver or release Rv was faster in acid pH (~5.0); a lower pH was more proximal to physiologic condition (~7.0); besides, its bio-safety was higher that others systems (93). *In vitro* and *in vivo* experiments analyze the brunt of the encapsulation of Rv on Am NPs plus human serum albumin (HSA), in PANC-1 cells and Balb/c nude mice. The procedure could encapsulate 62.5% of Rv and efficient drug release in pH 5.0 at 37°C; the system unleashed in most cells the cellular apoptosis (85%) by pyknotic nuclei formation; also the half-life time of the Np was improved with HAS at *in vivo* models. Furthermore the system didn’t have tissue toxicity (84). Another study demonstrated the apoptotic potential of Gc in human ovary cancer using Rv as a reducing and stabilizing agent in silver NPs; the system was conducted to a rate lower than 60% in the viability and cellular proliferation in A2780 line; likewise, it showed the free radical generation, and the treated cells raised the CASP3 and CASP9 expression. Furthermore the system conduced to DNA damage and the consecutive apoptosis induction (85). On the other hand, Gumireddy et al. generated a novel nano-compound based on 2-Hydroxypropyl *β*-cyclodextrin in complex with Cm and Rv in a solid lipid NP; this formulation increased the solubility of the NP. They confirmed that the bio-availability and the anticancer activity of the compounds had risen; *in vitro* application argued that drugs released were optimal in the physiological condition with an IC50 of 9.9 µM (94). The Rv effect in MDA-MB-231 BC cells promoted by the oxidized mesoporous carbon-Nps raised the intracellular levels of Rv in BC cells; the results pointed a 2.8 fold-change higher in relation with the control group; the apoptosis induction was 40% sharper in the control group; also they showed the compound caused apoptosis by the PAPR cleavage and activation of CASP3. Also the cytotoxicity of the Np was lower 24-h post-treatment (86). Another perspective demonstrated in primary patient samples of chronic lymphocyte leukemia the improved transfection of ribonucleic acids, such as mRNA and siRNA encapsulated in Nps mediated by Rv; this method proved the improvement of transfection rate after 1-h of exposition with 10 µM of Rv, putting that this approach enhances the transfection; besides, the results showed minimum toxicity rate in treated cells (95). For their part, Elgizawy et al. established that the nano-structuration of Rv inhibits cell proliferation and promoted apoptosis in various human cell lines treated with 12.5 µg/ml of Rv; the protector effect of the system was 33.96%; besides, the anti-tumor activity increased in the Hep-G2, HCT-116, 1301, and in MCF-7 cells 48-h post-treatment by the induction of various CASP such as CASP3, CASP6, CASP7 (96).

### Quercetin Nanoparticles in Cancer Treatment

Another attractive natural compound used in cancer therapy analysis is the Qr. **Table 3** summarizes some examples reported about the use of Qr Nps in the treatment of different cancer types.

**Table 3:** Examples of different nanoparticles loaded with Quercetin and their impact in the control of cancer.

Model	System	System size	Apoptosis induced by	C.P	T.G.	Ref.
Human neuroglioma	Gold nanoparticle	106.7 nm	Autophagosome formation and AKT/ERK/Caspase-3 pathway.	20%	N.V.T.	(97)
Breast cancer (Triple-negative)	Gold nanoparticles	4.5 nm	Nuclei Fragmentation, Bax and Bcl-2 regulation and suppression of PI3K.	48%	N.R.	(98)
Lung cancer	Bismuth selenide camouflaged in macrophage membrane	155.3 nm	Down-regulation of HSP70, inhibition of AKT, induction of CASP3 and the downregulation of metalloproteinase-9	56.7%	17%	(99)
Human colorectal cancer	1,2-distearoyl-sn-glycero-3-phosphoethanolamine-N-methoxy-poly plus D-α-tocopherol polyethylene glycol succinate	20 ± 0.6 nm	Inhibition of inflammatory molecules, increased of T-cell activity, and autophagy by the inhibition of mTOR and Bcl-2.	N.R	16.6%	(100)
Liver cancer	Chitosan-based in nano-hydrogel	912 nm	Increase of the DNA methylation and regulation of DNMTs.	20%	N.R	(101)

C.P., Cell Proliferation; T.G., Tumor Growth; N.R., Non-Reported; N.V.T, Non-Visible Tumor.

Bishayee et al. studied the Qr gold complex with Nps of poly (DL-lactide-co-glycolide); the treatment of HepG2 HCC, HeLa cells, A375 cells, and WRL-68 cells with the system showed differential noxious factors in cancer cells, especially in HepG2 cells; they proved the arrest in the S phase of the cell cycle, contributing to fewer cell proliferation. The complex has the capacity to interact with DNA and promote the production of ROS conducing the cell apoptosis (102). Other studies in HaCaT cell line treated with Poly (lactide-co-glycolide) copolymer loaded with Qr showed that the kinetics at physiological pH was similar in the acid pH, showing an accumulative drug release of 70% triggered a lower cancer’s cell viability rate in contrast with the control group (103). For its part a second report performed with the same Np system showed an encapsulation efficiency of 81.7% and the inhibition of COX-2 after 6-h with UV damage; besides, the capability of Qr as defensive factor increased in the face of at risk factors (104). Nano-diamonds loaded with Qr in HeLa cells showed that a concentration of 100 µM of Nps inhibited the cell growth in 54% 58-h post-treatment, and the anti-proliferative proprieties appeared in 74% of the cells; besides, the cell viability reduced to 50%, 72-h post-treatment; the results indicated the induction of apoptosis by the cleavage of pro-form CASP3 72-h post-treatment (105). Other repors revealed that Qr-gold Nps impacted in the autophagy induction and apoptosis in U87 cells, and in male BALB/c- mice, the use of 50 µg/ml reduced the cell viability up to 50%; still, *in vitro* experiments indicated conversion of LC3B-I in LC3B-II. Furthermore the p62 induction was reduced in the Qr treated group; meanwhile, the *in vivo* results showed a KI-67 decrease; besides, the mice cared for with the system didn’t develop detectable tumors, and the treatment could inhibit the PI3K/Akt/mTOR pathway (97). Fifty cervical cancer patient samples treated with NPs of gold loaded with Qr conduced to autophagosome induction and a poorer Janus Kinase 2 expression; besides, the treatment arrested at cells in phase G0/G1 and reduced the induction of S phase; this effect induced the down-regulation of STAT5 and Bcl-2 and upregulation of BAX, BAD, Cyto-c, Apaf-1 and CASP3. Furthermore, the results showed the suppression of the PI3K/AKT pathway, and the cyclin-D1 suppression led to the formation of auto-phagosomes and cell apoptosis (106). Other strategies based on super-paramagnetic Nps for cancer therapy enriched with Qr showed in the MDA-MB-231line and HeLa cells line that the system could load 12.1% of the drug and the encapsulated percentage was up to 80% of Qr; in physiologic conditions it showed that up to 83% of Qr could be released, 250-h post-treatment, triggering reduction in cell growth. Furthermore the nanocarrier gets an up bio-compatibility and strengthened the Qr intracellular delivery (107). In the context of specific site drug-delivery, Liu et al. reported that polymeric microspheres filled with Px and Qr were viable to treat LC; the method showed a 92.6% of encapsulating efficiency for Px and 90.3% for Qr; *in vitro* results showed that the drug-delivery, 2-h post-treatment at physiological condition was 21.87% for Px and 27.83% for Qr; besides, *in vivo* data showed that half-life of Qr *in situ* was 3.58-h and less than 5% erythrocytes damaged demonstrated high bio-safety of the compound (108). For their part, Balakrishnan et al. documented the effects of gold Nps loaded with Qr in MCF-7 and MDA-MB-231 lines; their results showed 75% Qr binds in the Np; likewise, 53% could release at acid pH and 16% at physiologic pH, reporting that 50 µM induced the reduction of cell viability. They reported the fragmented nuclei and the activation of apoptosis in 52% of cells, seemingly by the upregulation of Bax and the concomitant down-regulation of Bcl-2. The treated cells showed the slackening of phosphorylation at EGF receptor and the suppression of PI3K/Akt pathway (98). On the other hand, Ren et al. treated MHCC97H cell lines with Qr gold Nps showing lower cellular migration, the downregulation in c-Myc, cyclin-D1, CDK1, MMP7 and *β*-catenin genes, and the up-regulation of P-27. Furthermore the cleavage of CASP3, CASP9 besides the changes of Cytochrome-c localization increased 20% the apoptosis rate and regulated AP-2*β*/hTERT signaling, and p50/NF-*κ*B/COX-2 and Akt/ERK1/2 pathway; consistently, mouse models treated showed the tumor volumes and weights were significantly lower (109). Gold nano-cage with tetradecanol was loaded with Qr and Dx to analyze the co-delivery in MCF-7/ADR cells determining that the system could release 10% of Dx and 7% of Qr after 2-h at 37°C, showing a relation between the temperature and the release efficiency; besides, the treatment inhibited the expression of permeability glycoprotein in MCF-7/ADR cells; likewise, the rate of early apoptosis was 55.9%, and system arrested 57.9% cells in G2/M cell cycle phase in treated cells; besides the IC50 of the system was 1.5 µg/ml (110). Zhao et al. camouflaged Qr-loaded hollow bismuth selenide Nps in macrophage membrane for BC therapy; the system presented rapid binding and drug-releasing, showing a high bio-compatibility in 4T1 cells; *in vitro* data showed the down-regulation of HSP70, 43.3% apoptosis rate mediated by the AKT phosphorylation inhibition; the induction of cleaved CASP3 and photo-thermal therapy synergy in mouse model proved a strong ability of targeting after 6-h post-intravenous injection and the tumor size reduction presented 8 days post-treatment; interestingly, the metastasis capacity decreased at 17%, and the system presented a low hemolysis rate indicating high bio-compatibility (99). Different perspectives of treatment were approached by Nan’s research group that produced TPP Chitosan Nps in complex with Qr to treat and prevent skin deterioration and skin cancer in HaCaT cells and by topical application on mice models; *in vitro* data revealed that the system increased the internalization and retention in HaCaT skin cells; *in vivo* results confirmed the defensive effect of the system after UV damage by the inhibition of NF-*κ*B/COX-2 signaling pathway by downregulation of IkB-*α*. In fact, the system prevented the mice from developing edema in the experimental group, showing a higher thickness of epidermis and dermis (111). The therapy with 1,2-distearoyl-sn-glycero-3-phosphoethanolamine-N-methoxy-poly (ethylene glycol 2000) and D-*α*-tocopherol polyethylene glycol succinate in complex with Qr and alantolactone released 7.6% of total Qr loaded, showing in CT26-FL3 tumor-bearing mice that the treatment had a considerably smaller volume; the therapy reduced the content of Treg cells, showing the inhibition of IL-10, TGF-*β*, IL-1*β*, and CCL2, and the increasing the effect of CD3+ T-cells, and the system improved the survival rate (100). On the other hand, Liu et al. analyze the relation between nanocrystal with different sizes loaded with Qr and its biological effects. The A549 cell treatment with three different concentration systems (200 nm, 500 nm, and 3 µm) reduced 50% cellular proliferation, specifically in the 200 nm and 500 nm size systems; smaller nanocrystals with higher Qr concentrations correlated with a poorer formation of the microfilaments, blocking the normal localization of the actin fibers, and the reduction of STAT3 expression changes the migration rate after 24-h treatment (112). Other therapy perspectives based on Zr-MOF loaded with Qr could sensitize the DNA in different tumoral cell lines; the treatment triggered an 18% at the survival rate, and was more sensible to irradiation. The DNA breaking and the induction of *γ*-H2AX was higher in the treated group; *in vivo* models showed 8% of the bio-distribution *in situ*. The inhibition of HIF1 could suppress the development of neo-vascularization in tumor tissues, and the analysis of BALB/c mice showed that the treated group has a 52.8% tumor inhibition rate by the downregulation of Ki6 (113). In relation with the effect of Qr on DNA, Abbaszadeh et al. informed that use of chitosan-based in nano-hydrogel loaded with Qr could alter genomic global DNA methylation and down-regulate DNMTs (DNMT1/3A/3B) in HepG2 cancer cells, increasing the level of methylated cytosine, and the correlation between the use of Qr and DNA methylation rate improved the anti-tumor effect (101). Other analyses proved the Qr delivery by TiO2 and Al2O3 Nps in MCF-7 cells, showing that the use of 25 µg/ml of the system has 90% bio-compatibility, and the nanocarrier internalized plus irradiation reduced the cell viability at 50% in relation with the control group, Furthermore the treatment with nano-sheets plus irradiation promoted the ROS production, DNA breaking, and altered the functionalities of mitochondria triggering the apoptosis or other cell death pathways (114). Other processes described for producing economic Nps enriched with HA plus Qr for targeting tumor showed 100% compound release rate and promoted the internalization by the CD44 receptor in 4T1 cells and HepG2 cells; besides, they showed 26.55% apoptosis rate, and *in vivo* data showed that the treatment led to less tumor growth (115).

## Conclusions

Multiple evidence results of *in vitro*, *in vivo*, and of different phases of clinical studies emphasize the perspectives about the potential of natural compounds to apply such as drugs by cancer therapy; here we brought to light the general benefits of these compounds in face of synthetic drugs; besides, it showed up the role of the natural particles as an adjuvant in the minimization of the secondary effects in classical therapies as chemotherapy and surgery in diverse studies, and these results showed the improvement in the treated group with natural compounds. In this review, we focused especially on resveratrol and quercetin effects in cancer treatment. We noted an upsurge in interest of investigators to find out the mechanisms of compounds in the cancer treatment, and because of the report of different investigations, the possible specificity of these natural compounds in the sensitization of the abnormal cells that triggered different molecules that conduced to cellular damage by the activation of apoptosis, the induction of this pathway could be related by the activation of other pathways such as caspases, activation of PI3/AKT/mTOR and DNA double-strand break. The development of different techniques boosted the efficiency of the drug release; these technological advances could improve the treatment of cancer by the induction of immune system response; this viewpoint about nanomaterials complexing with different cellular membranes could emphasize the specificity of the drug by cancer cells’ treatment. In this understanding, the developer of nanomaterials mediated by *in silico* applications results in pre-designed system of molecules more efficient for certain types of cancers, potentiating the multiple benefits of nano-complex; likewise the novel approaches and modification of the classic methodologies could decrease the cost of these nanoparticle production. Despite the promising benefits presented in models *in vitro* and *in vivo* about the application of the resveratrol and quercetin complexing with different nano-materials enriched with certain drugs, and because of the insufficient clinical evidence, clinical studies in phase I are currently required to confirm the results referred in models *in vitro* and *in vivo*; besides, other technologies could simplify the bridge between these pre-clinical studies and clinical phase I studies, such the 3D culture analysis. Novel perspectives to prevent the evolution of any type of cancer are carried out centralized in the natural drugs’ treatment. The evidence presented in certain research proposed that a defensive character of resveratrol and quercetin could convert them such as in molecules’ potential application in preventive medicine; some data showed the preventive aspect of certain types of diets under a specific plan. The fast advancement in phytopharmaceutical investigation triggered many expansions to reinterpret the treatment for a disease, such as cancer, as complex. The many advantages of natural compounds use have been shown in different cancer types, which frequently have been proven in clinical studies in different phases, which although not concentrated on the resveratrol or quercetin complexing with any type of nano-material, they have showed that the use of these molecules can enhanced because this type of nano-formulations favors not only the treatment, but can be used as preventive complexes and like promising molecules and with hopeful results in the theranostic, thus improving the probabilities of the good progression of the patients.

## Author Contributions

MS-L, EJ-A, CL-C, AP-G, CL-P, and MS-C wrote all the sections of the manuscript. The Correspondence author and Principal author conceived and designed the review. All authors contributed to the article and approved the submitted version.

## Conflict of Interest

The authors declare that the research was conducted in the absence of any commercial or financial relationships that could be construed as a potential conflict of interest.
